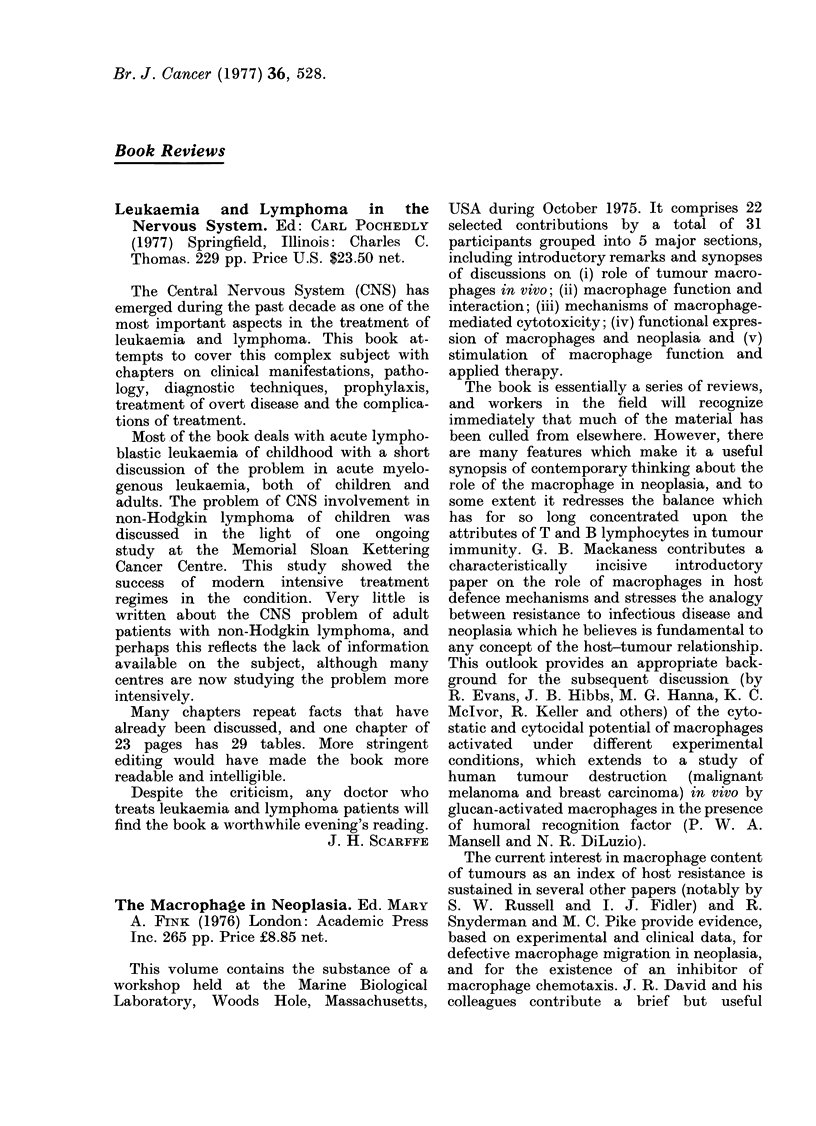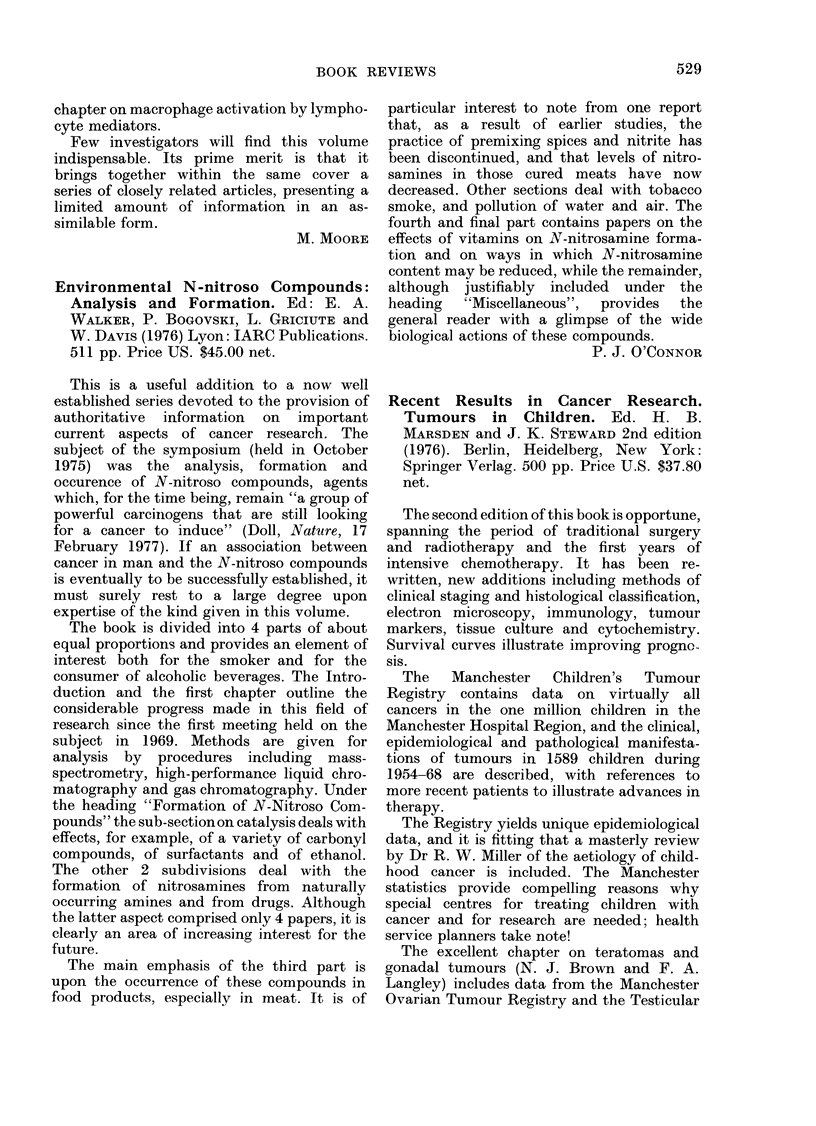# The Macrophage in Neoplasia

**Published:** 1977-10

**Authors:** M. Moore


**The Macrophage in Neoplasia.** Ed. MARY A. FINK (1976) London: Academic Press Inc. 265 pp. Price £8.85 net.

This volume contains the substance of a workshop held at the Marine Biological Laboratory, Woods Hole, Massachusetts, USA during October 1975. It comprises 22 selected contributions by a total of 31 participants grouped into 5 major sections, including introductory remarks and synopses of discussions on (i) role of tumour macrophages *in vivo*; (ii) macrophage function and interaction; (iii) mechanisms of macrophage-mediated cytotoxicity; (iv) functional expression of macrophages and neoplasia and (v) stimulation of macrophage function and applied therapy.

The book is essentially a series of reviews, and workers in the field will recognize immediately that much of the material has been culled from elsewhere. However, there are many features which make it a useful synopsis of contemporary thinking about the role of the macrophage in neoplasia, and to some extent it redresses the balance which has for so long concentrated upon the attributes of T and B lymphocytes in tumour immunity. G. B. Mackaness contributes a characteristically incisive introductory paper on the role of macrophages in host defence mechanisms and stresses the analogy between resistance to infectious disease and neoplasia which he believes is fundamental to any concept of the host–tumour relationship. This outlook provides an appropriate background for the subsequent discussion (by R. Evans, J. B. Hibbs, M. G. Hanna, K. C. McIvor, R. Keller and others) of the cytostatic and cytocidal potential of macrophages activated under different experimental conditions, which extends to a study of human tumour destruction (malignant melanoma and breast carcinoma) *in vivo* by glucan-activated macrophages in the presence of humoral recognition factor (P. W. A. Mansell and N. R. DiLuzio).

The current interest in macrophage content of tumours as an index of host resistance is sustained in several other papers (notably by S. W. Russell and I. J. Fidler) and R. Snyderman and M. C. Pike provide evidence, based on experimental and clinical data, for defective macrophage migration in neoplasia, and for the existence of an inhibitor of macrophage chemotaxis. J. R. David and his colleagues contribute a brief but useful chapter on macrophage activation by lymphocyte mediators.

Few investigators will find this volume indispensable. Its prime merit is that it brings together within the same cover a series of closely related articles, presenting a limited amount of information in an assimilable form.

M. MOORE